# Compartment Niche Shapes the Assembly and Network of *Cannabis sativa*-Associated Microbiome

**DOI:** 10.3389/fmicb.2021.714993

**Published:** 2021-10-05

**Authors:** Guangfei Wei, Kang Ning, Guozhuang Zhang, Haibin Yu, Shuming Yang, Fei Dai, Linlin Dong, Shilin Chen

**Affiliations:** ^1^Key Laboratory of Beijing for Identification and Safety Evaluation of Chinese Medicine, Institute of Chinese Materia Medica, China Academy of Chinese Medical Sciences, Beijing, China; ^2^Yunnan Industrial Investment Group, Yunnan Hemp Seed Industry Co., Ltd., Kunming, China

**Keywords:** HEMP, host selection, compartment niche, community assembly, transmission model

## Abstract

Interactions between plants and microbes may promote the growth of plants and regulate the production of secondary metabolites. Hemp (*Cannabis sativa*) is an annual herb and an important commercial crop. However, the assembly and network of hemp-associated microbiomes inhabiting in soil and plant compartments have not been comprehensively understood. This work investigated the assembly and network of bacterial and fungal communities living in soils (bulk and rhizosphere) and plant compartments (root, stem, leaf, and flower) of four hemp ecotypes cultivated in the same habitat. Microbiome assembly was predominantly shaped by compartment niche. Microbial alpha diversity was the highest in soil, continually decreased from root to flower. Core bacterial genera *Pseudomonas*, *Bacillus*, *Rhizobium*, *Planococcus*, and *Sphingomonas* were mostly enriched in aerial endosphere niches; *Clitopilus*, *Plectosphaerella*, and *Mortierella* were enriched in belowground endosphere. Microbial network complexity and connectivity decreased from root to flower. According to source tracking analysis, hemp microbiota primarily originated from soil and were subsequently filtered in different plant compartments. This work provides details on hemp-associated microbiome along the soil–plant continuum and a comprehensive understanding of the origin and transmission mode of endophytes in hemp.

## Introduction

Plant-associated microbiome, which comprises diverse microbial classes, such as bacteria, archaea, fungi, and oomycetes (Andrews and Harris, 2000), is considered as the second genome of host plant and varies among plant species (Bouffaud et al., 2014; Brown et al., 2020). These microbial communities play key roles in keeping soil homeostasis and improving host productivity through many beneficial activities, such as promoting plant growth (Gourion et al., 2016), protecting against pathogens (Innerebner et al., 2011), and producing secondary metabolites (Weyens et al., 2009).

The niche differentiation of microbial communities between rhizosphere soil and endosphere niches has been extensively studied (Lundberg et al., 2012). Compared with endosphere bacterial and fungal communities from *Populus deltoides*, the counterparts in rhizosphere exhibited lower inter-sample variability (Gottel et al., 2011) and higher biodiversity (Beckers et al., 2017). The differences in diversity and composition of the microbiome obtained from different plant compartments have also been reported. For sugar maple (*Acer saccharum*), bacterial and fungal communities varied among four compartments (i.e., leaf epiphytes, leaf endophytes, root epiphytes, and root endophytes) (Wallace et al., 2018). For tomato (*Solanum lycopersicum*), roots have substantially enriched microbial diversity compared with the aerial parts (i.e., bottom leaf, stem, fruit, flower, and top leaf) (Ottesen et al., 2013). Each plant compartment possesses a highly distinct microbial community and provides a unique ecological niche (Wei and Ashman, 2018). Therefore, elucidating the origin and transmission mode of plant microbiomes is pivotal to plant agricultural practices.

Soil environment is widely considered as a hotspot for studying the biodiversity and origin of plant-associated microbiomes (Compant et al., 2010). The transmission mode of plant microbiomes involves two different pathways (i.e., horizontal transmission and vertical transmission) (Wei and Ashman, 2018). The horizontal transmission model suggests that soil-borne microbiome in the bulk soil first transfers to the rhizosphere soil, then concentrates around the rhizoplane of root tissues, and finally colonizes the inner parts of root tissues (Compant et al., 2016). The vertical model involves the transmission of rhizosphere microflora to the aerial parts of the host (i.e., stem, leaf, flower, fruit, and seed) as endophytes (Compant et al., 2010). Endophytes are microbial communities that colonize and coexist harmoniously in the healthy tissues of plants (Compant et al., 2011). In most plants, a higher number of microbial species is observed in the belowground parts than that in the aboveground parts because only a small fraction of microbes can be preserved during vertical transmission (Koiv et al., 2015). Additionally, genetic variation is ubiquitous in most host plants and may affect the microbiome composition (Wagner et al., 2016); however, limited information is available about the influences of multiple genotypes on microbiomes. Research concerning multiple genotypes is thus crucial to reveal the roles of plant genotypes in modulating microbiota construction.

*Cannabis sativa* is an annual herb and cultivated worldwide as hemp (THC < 0.3%) or marijuana (THC > 0.3%). Hemp, which has been cultivated and used for 5,000–6,000 years, has attracted attention because of its production of fiber, seed, and oil (Bonini et al., 2018; Russo et al., 2018). Hemp seed is a commonly used medicinal material and a good food source (Taghinasab and Jabaji, 2020). In view of the roles of microbes in promoting the growth and health of host plant, understanding the ecology of microbial communities is critical for the improvement of hemp industry. Gautam et al. (2013) found that the abundance of fungal endophytes in *C. sativa* varied across different geographical regions. The diversity and composition of rhizosphere microbial community of *C. sativa* were predominantly determined by soil types, and the community structure in endosphere was mainly shaped by host cultivars (Winston et al., 2014). The microbial composition of *C. sativa* across six fields was largely explained by plant compartments (Barnett et al., 2020). The fungal and bacterial microbiomes of *C. sativa* exhibited spatial–temporal and cultivar-dependent variations (Comeau et al., 2020). Punja et al. (2019) identified the response of different cannabis strains (genotypes) to various pathogens and found that diverse endophytes existed in different compartments. Studies on how the compartment niche of different hemp ecotypes shaped the microbiome assemblies and co-occurrence patterns, however, have rarely been conducted, hindering our ability to improve the agricultural practices of hemp by modulating microbiome (McKernan et al., 2020). Additionally, the origin and transmission mode of hemp-associated microbiome remain unknown.

In this work, the bacterial and fungal communities were characterized across 72 samples from soils (bulk and rhizosphere) and multiple compartment niches (root, stem, leaf, and flower) of four hemp ecotypes cultivated in the same controlled environment. We aim to: (1) evaluate how the microbiome assemblies and co-occurrence patterns in the bulk soil, rhizosphere soil, and endosphere are affected by compartment niches and host ecotypes; (2) identify differential taxa in each host niche and the potential sources of observed microbial communities. The following hypotheses are proposed: (1) the assemblies and network complexity of hemp-associated microbiome are mainly influenced by compartment niches; (2) plant microorganisms primarily originated from soil and were subsequently filtered from soils to endosphere samples.

## Materials and Methods

### Experimental Design and Sample Preparation

Four hemp ecotypes (THC < 0.3%) were investigated according to their different biological characteristics and types: Gansuqingshui (GS, seed type), Yunnan No. 1 (YN, seed and stem compatible type), Yunmaza No. 1 (MG, fiber, and medicine-compatible type), and Huoma No. 1 (HLJ, fiber type) (Supplementary Table 1). The soils were purchased from Shanghai Yiang Landscaping Co., Ltd., from China. All hemp plants were grown in the same soil (available N, 2,450 mg/kg; available P, 12.88 mg/kg; available K, 1,000 mg/kg; organic matter 374 mg/kg, cation exchange capacity 25.31 mol/kg; pH 6.5; Cu 46.30 mg/kg, Zn 549.26 mg/kg, Pb 84.38 mg/kg, Cd 3.54 mg/kg, and Ni 76.25 mg/kg), and cultivated in pots under controlled growth chamber with 16 h light (temperature, 28°C; humidity, 60%) and 8 h night (temperature, 20°C; humidity, 40%) for vegetative growth. After 60 days, the cycle was changed into 12 h light (temperature, 28°C; humidity, 60%) and 12 h night (temperature, 20°C; humidity, 40%) for reproductive growth (Dong et al., 2018a). The plants were grown without fertilization and watered twice a week.

Fifteen plants were collected per ecotype when the 85-day-old hemp plants were in full flower stage (Barnett et al., 2020). The soils away from the roots (10–20 cm) with a depth of 10 cm were collected as bulk soils (Ct). The soils attached to the roots (0–3 mm) were gently removed from roots and collected as rhizosphere soil samples (Rs). The above soil samples were carefully homogenized, sieved (2 mm) and stored at −80°C until DNA extraction. Hemp roots (Ro), stems (St), leaves (Le), and flowers (Fl) were randomly collected, carefully washed and surface-sterilized by conducting the following immersions: 70% (v/v) ethanol for 3 min, 2.5% (v/v) sodium hypochlorite (NaClO) for 5 min, and sterile water four times (Barra et al., 2016). Sterility test was performed to check whether the plant surface was sterilized cleanly (Carrión et al., 2019). The properly surface-sterilized samples were aseptically cut, rapidly frozen, ground to power, and stored at −80°C for DNA extraction.

### DNA Extraction, PCR, and Sequencing

Total DNA from 500 mg of each sample was extracted in accordance with the instructions of the FastDNA SPIN Kit for Soil (MoBio Laboratories, Inc., United States). DNA quality was detected by a NanoDrop 1000 spectrophotometer (Thermo Scientific, United States). The 16S rRNA gene fragments were nested amplified with three pairs of bacterial primers (5′-CCGCGTGNRBGAHGAAGGYYYT-3′)/(5′-TAATCCTGTTTGCTCCCCAC-3′), (5′-CCGCGTGNRB GAHGAAGGYYYT-3′)/(5′-GACTACHVGGGTWTCTAATC CTGTTTGCTC-3′), and (5′-GTGYCAGCMGCCGCGGTAA-3′)/(5′-GACTACHVGGGTATCTAATCC-3′), to effectively reduce the efficiency of chloroplast sequence amplification. The chloroplast sequence was not annotated from species identification inoculation (Yu et al., 2013). The obtained ITS rRNA gene fragments were amplified using two pairs of fungal primers, (5′-CTTGGTCATTTAGAGGAAGTAA-3′)/(5′-TCCTCCGCTTATTGATATGC-3′) and (5′-GTGART CATCGAATCTTTG-3′)/(5′-TCCTCCGCTTATTGATATGC-3′) (Yao et al., 2019). The PCR products were purified as previously described (Dong et al., 2018b). The DNA products were sequenced using Illumina HiSeq platform (Illumina, United States), and 250 bp paired-end reads were obtained.

### Data Processing and Statistical Analyses

The sequences were assigned to different samples based on their barcodes, and QIIME 2 software (v1.7.0) was used to obtain effective tags (Bolyen et al., 2019). Chloroplast operational taxonomic units (OTUs) and rare bacteria (<20 reads) were then removed (Laforest-Lapointe et al., 2016). OTU was defined as a cluster with 97% similarity level using USEARCH software (Dong et al., 2016). After read-quality filtering, 60,563,867 and 6,066,030 high-quality reads were obtained in 72 samples for 16S and ITS sequencing samples, respectively (Supplementary Table 2). The number of high-quality bacterial reads ranged from 61,945 to 125,752, and that of high-quality fungal reads ranged from 80,250 to 87,678. All raw sequencing data were deposited in the Sequence Read Archive (SRA) database of the National Center for Biotechnology Information (NCBI) with the accession numbers of PRJNA690686 (bacteria) and PRJNA690692 (fungi).

Non-metric multidimensional scaling (NMDS) ordination analyses based on Bray–Curtis, unweighted UniFrac, and weighted UniFrac distance were performed to investigate the variation patterns in microbial community among different compartments and ecotypes (Caporaso et al., 2010). The significance of compartment niches and host ecotypes on community dissimilarity was tested by the permutational multivariate ANOVA (PERMANOVA) using the “Adonis” function in *R* (Oksanen et al., 2007). Alpha diversity indices, including Chao 1 and Shannon index, were calculated using mothur program (Schloss et al., 2009). One-way ANOVA and Turkey’s honestly significant difference (HSD) tests were performed to measure significant differences in microbial diversity and composition among different compartment niches. Circos plots showing the distribution proportion of core microbiota were visualized using the Circos table viewer^1^ (Krzywinski et al., 2009). Linear discriminant analysis effect size (LEfSe) was applied to identify the biomarkers among different compartment niches (*p* < 0.05 and LDA score >4) (Segata et al., 2011). Co-occurrence analyses were performed based on Spearman’s correlation scores among microbial taxa. Only robust (Spearman’s *r* > 0.8, or *r* < −0.8), and statistically significant (*p* < 0.05) correlations were retained. Network visualization and property measurements were calculated with the interactive platform Gephi (Bastian et al., 2009). Source tracking analysis was conducted to calculate the proportion of endophytic bacteria and fungi in each host niche derived from soils (Knights et al., 2011).

## Results

### Microbial Beta Diversity Was Mainly Shaped by Compartment Niches, Not by Ecotypes

**FIGURE 1:**
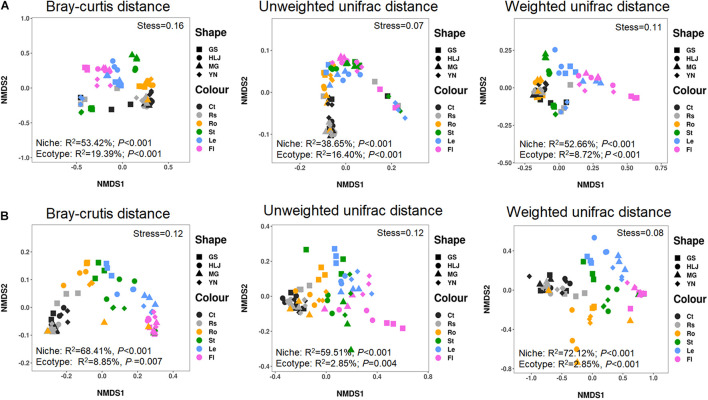
Non-metric multidimensional scaling (NMDS) ordinations of microbial communities associated with hemp plants. **(A)** Bacteria. **(B)** Fungi. GS, HLJ, MG, and YN represent Gansuqingshui, Huoma No. 1, Yunmaza No. 1, and Yunnan No. 1, respectively. Ct, Rs, Ro, St, Le, and Fl represent bulk soil, rhizosphere soil, root endosphere, stem endosphere, leaf endosphere, and flower endosphere, respectively.

The results showed that variations in bacterial community were predominantly affected by compartment niches (Bray–Curtis distance, *R*^2^ = 53.42%, *p* < 0.001; unweighted UniFrac distance, *R*^2^ = 38.65%, *p* < 0.001; weighted UniFrac distance, *R*^2^ = 52.66%, *p* < 0.001), not by host ecotypes (Bray–Curtis distance, *R*^2^ = 19.39%, *p* < 0.001; unweighted UniFrac distance, *R*^2^ = 16.40%, *p* < 0.001; weighted UniFrac distance, *R*^2^ = 8.72%, *p* < 0.001) (Figure 1A).

Variations in fungal community were also mainly explained by compartment niches (Bray–Curtis distance, *R*^2^ = 68.41%, *p* < 0.001; unweighted UniFrac distance, *R*^2^ = 59.51%, *p* < 0.001; weighted UniFrac distance, *R*^2^ = 72.12%, *p* < 0.001) and not by host ecotypes (Bray–Curtis distance, *R*^2^ = 8.85%, *p* = 0.007; unweighted UniFrac distance, *R*^2^ = 10.79%, *p* = 0.004; weighted UniFrac distance, *R*^2^ = 2.85%, *p* = 0.129). These results showed that variations in bacterial and fungal communities presented similar patterns and could be primarily explained by plant compartments in the same habitat (Figure 1B).

### Microbial Alpha Diversity Decreased From Soils to Endosphere Samples

**FIGURE 2:**
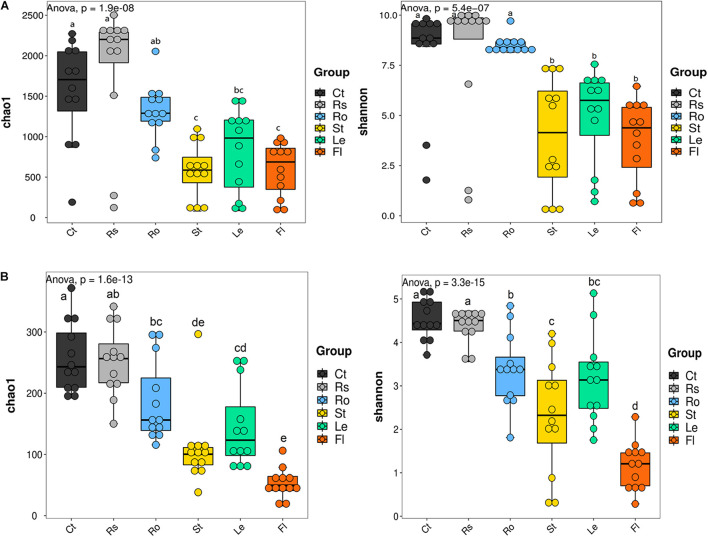
Alpha diversity of microbial communities associated with hemp plants. **(A)** Bacteria. **(B)** Fungi. Ct, Rs, Ro, St, Le, and Fl represent bulk soil, rhizosphere soil, root endosphere, stem endosphere, leaf endosphere, and flower endosphere, respectively.

The alpha diversity of microbial communities was calculated to further assess the effect of compartment niches on hemp-associated microbiomes. Significant differences in the alpha diversity of bacterial community were observed among different compartments (*p* < 0.001; Figure 2A and Supplementary Table 3). Bacterial Chao 1 was significantly greater in soils (bulk soil, 1,560.73 ± 624.99; rhizosphere soil, 1,845.69 ± 807.81) and root endosphere samples (1,315.16 ± 343.50) than in aerial endosphere samples (stem endosphere, 581.42 ± 340.48; leaf endosphere, 829.01 ± 509.35; flower endosphere, 595.70 ± 330.34). Bacterial Shannon value was significantly higher in soil and root endosphere samples (bulk soil, 8.08 ± 2.61; rhizosphere soil, 8.08 ± 3.42; root endosphere, 8.52 ± 0.44) than in aerial endosphere samples (stem endosphere, 3.99 ± 2.85; leaf endosphere, 4.95 ± 2.38; flower endosphere, 3.77 ± 2.04).

Fungal alpha diversity was also strongly influenced by compartment niches (*p* < 0.001; Figure 2B and Supplementary Table 3). Fungal alpha diversity was the highest in bulk soil (Chao 1, 258.03 ± 57.61; Shannon, 4.53 ± 0.47), and rhizosphere soil (Chao 1, 252.57 ± 59.94; Shannon, 4.38 ± 0.39) and incrementally decreased from root endosphere samples (Chao 1, 186.35 ± 66.33; Shannon, 3.38 ± 0.85) and stem endosphere samples (Chao 1, 108.93 ± 63.19; 2.32 ± 1.31), to leaf endosphere samples (Chao 1, 144.76 ± 66.94; Shannon, 3.15 ± 1.01) and finally to flower endosphere samples (Chao 1, 53.14 ± 24.21; Shannon, 1.15 ± 0.56). These results showed that microbial alpha diversity was the highest in bulk soil and rhizosphere soil samples, gradually decreased from root endosphere samples to flower endosphere samples.

### Different Compositions of Core Microbiota Among Soils and Endosphere Niches

**FIGURE 3:**
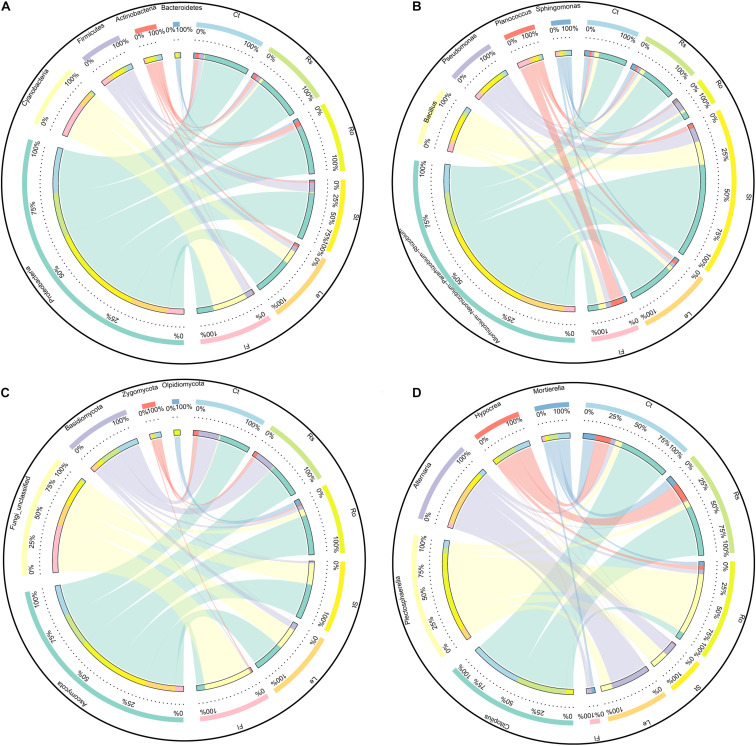
Circos plot showing the distribution proportion of core microbiota among plant compartments. **(A)** Core bacterial phyla. **(B)** Core bacterial genus. **(C)** Core fungal phyla. **(D)** Core fungal genus. Ct, Rs, Ro, St, Le, and Fl represent bulk soil, rhizosphere soil, root endosphere, stem endosphere, leaf endosphere, and flower endosphere, respectively.

The flower plot of bacterial community showed 1,006, 1,046, 872, 731, 933, and 781 OTUs specifically distributed in bulk soil, rhizosphere soil, root endosphere, stem endosphere, leaf endosphere, and flower endosphere samples, respectively (Supplementary Figure 1A). These OTUs were assigned to different taxonomic levels to further examine the exact composition of bacterial community in different compartments (Figure 3A and Table 1). At the bacterial phylum level, Proteobacteria (67.26%), Cyanobacteria (14.40%), Firmicutes (8.07%), Actinobacteria (4.93%), and Bacteroidetes (1.49%) were the top five phyla and presented significant compartment-specificity (*p* < 0.05) except for Firmicutes (*p* = 0.111). Cyanobacteria and Firmicutes had the greatest proportion in the flower endosphere samples, and Proteobacteria, Actinobacteria, and Bacteroidetes were mostly enriched in soil and root endosphere samples. At the genus level, *Rhizobium* (16.85%), *Pseudomonas* (3.14%), *Planococcus* (1.99%), *Bacillus* (1.73%), and *Sphingomonas* (1.34%) were the top five genera and showed significant compartment-specific (*p* < 0.05) (Figure 3B and Table 1). The highest relative abundance of *Pseudomonas* and *Bacillus* were in the flower endosphere samples, whereas the highest abundance of *Rhizobium* and *Sphingomonas* were in the stem endosphere samples.

**TABLE 1:** The top five most abundant bacterial communities within compartments in hemp plants (*x* ± SD).

	Compartments	Bulk soil (%)	Significant difference	Rhizo sphere soil (%)	Significant difference	Root endo sphere (%)	Significant difference	Stem endo sphere (%)	Significant difference	Leaf endo sphere (%)	Significant difference	Flower endo sphere (%)	Significant difference	*F*(5,71)	*p*-Value
Phylum	Proteobacteria	75.04 ±7.21	ab	74.32 ±10.63	ab	77.83 ±5.35	a	85.18 ±10.92	A	63.07 ±21.69	b	28.14 ±16.30	C	28.71	0.000
	Cyanobacteria	1.05 ±0.30	c	4.01 ±0.79	c	0.35 ±0.56	c	1.16 ±0.85	c	22.27 ±16.40	b	57.57 ±24.97	A	38.55	0.000
	Firmicutes	8.63 ±4.48	ab	6.79 ±3.24	b	4.07 ±2.79	ab	8.71 ±4.88	ab	8.32 ±5.16	ab	11.90 ±10.84	A	1.88	0.111
	Actinobacteria	6.58 ±2.84	ab	6.08 ±3.26	abc	8.58 ±4.32	a	3.22 ±2.70	cd	3.82 ±2.70	bcd	1.28 ±1.03	D	9.47	0.000
	Bacteroidetes	1.81 ±1.35	b	1.81 ±1.23	b	3.55 ±0.69	a	0.76 ±0.69	c	0.63 ±0.41	c	0.35 ±0.26	C	22.40	0.000
Genus	*Rhizobium*	13.05 ±6.52	ab	15.92 ±2.90	ab	3.03 ±1.40	b	43.18 ±13.15	a	21.07 ±10.38	ab	4.83 ±5.85	B	2.93	0.019
	*Pseudomonas*	0.97 ±0.87	c	3.98 ±2.28	c	0.33 ±0.24	c	1.15 ±0.85	c	22.25 ±4.73	b	57.56 ±7.21	A	38.58	0.000
	*Planococcus*	1.33 ±0.15	b	1.72 ±0.28	b	4.86 ±0.85	b	6.57 ±1.93	ab	1.86 ±0.56	a	2.52 ±0.67	B	4.97	0.001
	*Bacillus*	0.99 ±0.37	b	0.91 ±0.59	b	0.68 ±0.12	b	1.90 ±0.56	b	1.58 ±0.57	b	5.91 ±1.58	A	6.46	0.000
	*Sphingomonas*	1.08 ±0.24	b	0.91 ±0.13	b	0.60 ±0.15	b	3.98 ±1.17	a	1.05 ±0.26	b	2.76 ±1.39	ab	3.111	0.014

For ITS sequences, 220 OTUs were separately identified in bulk soil compared with rhizosphere soil (213), root endosphere (234), stem endosphere (262), leaf endosphere (287), and flower endosphere samples (146) (Supplementary Figure 1B). Ascomycota (50.59%) was the most abundant phylum, followed by Fungi_unclassified (28.45%), Basidiomycota (15.43%), Zygomycota (3.13%), and Olpidiomycota (1.68%) (Figure 3C and Table 2). These five fungal phyla displayed a significant compartment specificity (*p* < 0.05). Among which, Ascomycota had the lowest relative abundance in the flower endosphere samples, and Basidiomycota and Zygomycota exhibited the highest relative abundance in the soil samples. Significant compartment effects were also observed in the top fungal genera *Clitopilus* (11.90%), *Plectosphaerella* (11.26%), *Alternaria* (7.35%), *Hypocrea* (4.36%), and *Mortierella* (3.07%) (*p* < 0.05). *Clitopilus*, *Plectosphaerella*, and *Mortierella* were significantly enriched in the bulk soil, rhizosphere soil, and root endosphere samples compared with those of the aerial parts (*p* < 0.05; Figure 3D and Table 2). These results showed that the soil and plant compartment samples shared common core species but differed in structural composition.

**TABLE 2:** The top five most abundant fungal communities within compartments in hemp plants (*x* ± SD).

	Compartments	Bulk soil (%)	Significant difference	Rhizo sphere soil (%)	Significant difference	Root endo sphere (%)	Significant difference	Stem endo sphere (%)	Significant difference	Leaf endo sphere (%)	Significant difference	Flower endo sphere (%)	Significant difference	*F*(5,71)	*p*-Value
Phylum	Ascomycota	52.77 ±13.81	a	57.05 ±15.83	a	68.45 ±26.18	a	59.90 ±34.97	A	51.27 ±19.36	a	14.11 ±4.74	B	8.732	0.000
	Fungi_unclassified	2.12 ±0.23	c	1.10 ±0.66	c	11.45 ±2.15	c	36.62 ±6.56	b	37.36 ±21.61	b	82.02 ±15.38	a	27.42	0.000
	Basidiomycota	35.72 ±15.50	a	34.15 ±14.92	a	6.78 ±3.65	b	3.17 ±0.53	b	10.88 ±5.98	b	1.88 ±0.24	b	28.93	0.000
	Zygomycota	8.11 ±4.50	a	6.19 ±2.57	a	2.27 ±0.35	b	0.18 ±0.42	b	0.08 ±0.02	b	1.93 ±0.64	b	9.30	0.000
	Olpidiomycota	0.66 ±0.32	b	0.23 ±0.06	b	8.97 ±7.67	a	0.06 ±0.11	b	0.12 ±0.03	b	0.05 ±0.03	b	15.42	0.000
Genus	*Clitopilus*	33.63 ±16.55	a	32.82 ±13.61	a	4.68 ±2.95	b	0.08 ±0.05	b	0.04 ±0.02	b	0.17 ±0.09	b	35.25	0.000
	*Plectosphaerella*	4.57 ±1.76	b	4.05 ±1.54	b	40.57 ±7.48	a	10.95 ±4.27	b	7.21 ±3.59	b	0.24 ±0.07	b	14.18	0.000
	*Alternaria*	3.29 ±1.05	b	0.96 ±0.61	b	2.89 ±0.81	b	8.27 ±2.55	b	25.69 ±3.64	a	2.98 ±1.00	b	22.76	0.000
	*Hypocrea*	10.27 ±1.02	a	12.32 ±2.28	a	2.95 ±1.08	b	0.34 ±0.08	b	0.22 ±0.07	b	0.08 ±0.04	b	24.57	0.000
	*Mortierella*	7.94 ±1.27	a	6.05 ±0.73	a	2.23 ±1.10	b	0.17 ±0.12	b	0.07 ±0.04	b	1.93 ±1.87	b	9.10	0.000

### Microbial Biomarkers Obtained Among Hemp-Associated Microbiome

**FIGURE 4:**
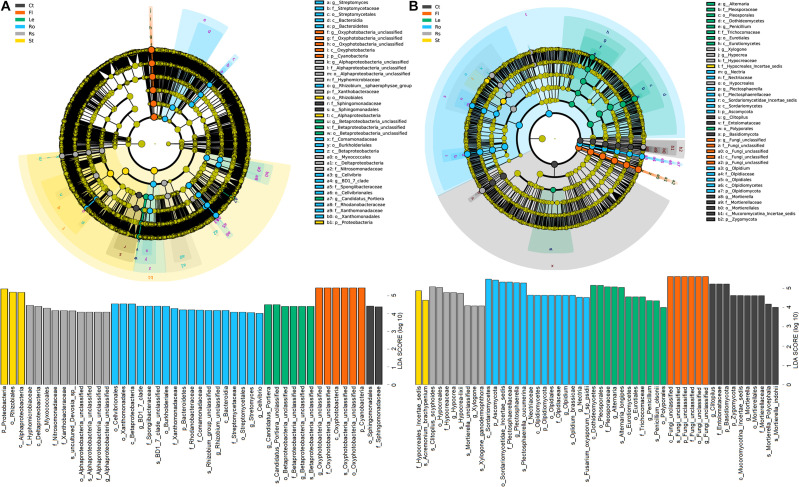
Linear discriminant effect size (LEfSe) of bacterial **(A)** and fungal **(B)** communities associated with hemp plants with a linear discriminant analysis (LDA) score higher than 4.0 and *p*-values less than 0.05. Ct, Rs, Ro, St, Le, and Fl represent bulk soil, rhizosphere soil, root endosphere, stem endosphere, leaf endosphere, and flower endosphere, respectively.

Linear discriminant analysis effect size revealed differences in community composition among soils (bulk soil and rhizosphere soil) and plant endosphere samples (root, stem, leaf, and flower endospheres) (Figure 4). Among the 45 bacterial biomarkers (LDA > 4), 2, 10, 18, 3, 6, and 6 were enriched in the bulk soil, rhizosphere soil, root endosphere, stem endosphere, leaf endosphere, and flower endosphere, respectively (Figure 4A). The order Sphingomonadales and family Sphingomonadaceae were enriched in the bulk soil. The classes Alphaproteobacteria and Deltaproteobacteria, the order Myxococcales, and the families Hyphomicrobiaceae, Nitrosomonadaceae, and Xanthobacteraceae were enriched in the rhizosphere soil. The classes Betaproteobacteria and Bacteroidia, the orders Cellvibrionales, Xanthomonadales, Burkholderiales, and Streptomycetales, the families Spongiibacteraceae, Xanthomonadaceae, Rhodanobacteraceae, Comamonadaceae, and Streptomycetaceae, and the genera *Rhizobium*, *Streptomyces*, and *Candidatus–Portiera* were enriched in the root endosphere. The phylum Proteobacteria, the class Alphaproteobacteria and the order Rhizobiales were enriched in the stem endosphere. The phylum Cyanobacteria and the class Oxyphotobacteria were enriched in the flower endosphere.

Among the 52 fungal biomarkers (LDA > 4.0), 10, 8, 15, 2, 11, and 6 taxa were enriched in the bulk soil, rhizosphere soil, root endosphere, stem endosphere, leaf endosphere, and flower endosphere, respectively (Figure 4B). The phyla Basidiomycota and Zygomycota, the order Mortierellales, the families Entolomataceae and Mortierellaceae and the genera *Clitopilus* and *Mortierella* were enriched in the bulk soil. The family Hypocreaceae and genera *Hypocrea* and *Xylogone* were enriched in the rhizosphere soil. The phyla Ascomycota and Olpidiomycota, the order Olpidium, the families Plectosphaerellaceae, Nectriaceae, and Olpidiaceae and the genera *Plectosphaerella*, *Olpidium*, and *Nectria* were enriched in the root endosphere. The classes Dothideomycetes and Eurotiomycetes, the orders Pleosporales, Eurotiales, and Polyporales, the families Pleosporaceae and Trichocomaceae, and the genera *Alternaria*, and *Penicillium* were enriched in the stem endosphere.

### Microbial Network Complexity and Connectivity Decreased From Root Endosphere to Flower Endosphere

**FIGURE 5:**
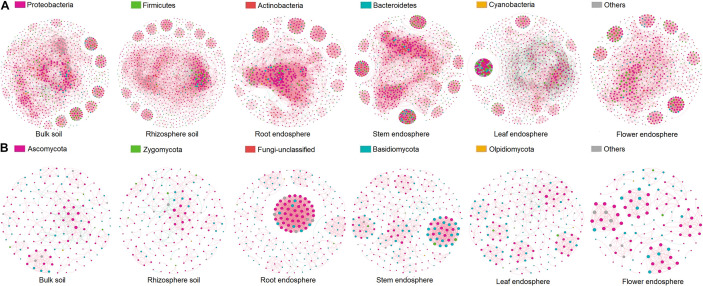
Co-occurrence network analysis of microbial communities associated with hemp plants. **(A)** Bacteria. **(B)** Fungi.

The co-occurrence patterns of microbial communities among soils and plant compartments showed the influence of compartment niches on microbial network complexity (as indicated by average degree) and connectivity. In the co-occurrence network of bacterial communities, the average degree was the greatest in root endosphere (30.08), followed by that in stem endosphere (28.90), and leaf endosphere (20.43), and was the lowest in flower endosphere (16.91) (Figure 5A and Table 3). Higher values of topological properties (i.e., nodes, edges, positive edges, average clustering coefficient, and total triangles) were detected in the root endosphere (993, 13,480, 13,431, 0.74, and 128,440, respectively) than those in stem, leaf, and flower endosphere niches (stem: 781, 10,993, 10,910, 0.70, and 133,388, respectively; leaf: 872, 8,908, 7,494, 0.68, and 114,124, respectively; flower: 731, 6,602, 6,516, 0.66, and 39,763, respectively).

**TABLE 3:** Topological properties of co-occurring bacterial and fungal networks within hemp compartments.

	Network properties	Bulk soil	Rhizosphere soil	Root endosphere	Stem endosphere	Leaf endosphere	Flower endosphere
Bacteria	Number of nodes	1,006	1,046	993	781	872	731
	Number of edges	9,304	10,255	13,480	10,993	8,908	6,602
	Positive edges	9,218	9,963	13,431	10,910	7,494	6,516
	Negative edges	86	292	83	49	1,414	86
	Modularity	0.83	0.78	0.63	0.80	0.80	0.83
	Number of communities	72	63	53	45	88	66
	Network diameter	11	15	13	10	10	13
	Average path length	4.018	4.18	3.31	3.68	3.85	3.92
	Average degree	18.50	19.61	30.08	28.90	20.43	16.91
	Average clustering coefficient	0.64	0.60	0.74	0.70	0.68	0.66
	Density	0.018	0.019	0.041	0.031	0.023	0.022
	Total triangles	54,594	69,969	128,440	133,388	114,124	39,763
Fungi	Number of nodes	160	159	216	230	189	117
	Number of edges	384	340	1,670	1,102	546	336
	Positive edges	335	299	1,664	1,089	522	365
	Negative edges	49	41	6	13	24	1
	Modularity	0.80	0.75	0.41	0.77	0.85	0.86
	Number of communities	24	30	30	32	27	23
	Network diameter	12	13	12	9	14	4
	Average path length	3.94	4.18	1.78	2.32	4.28	1.21
	Average degree	5.80	6.28	15.46	9.58	6.26	5.78
	Average clustering coefficient	0.72	0.75	0.88	0.86	0.71	0.72
	Density	0.03	0.03	0.07	0.04	0.03	0.05
	Total triangles	564	400	21,599	5,941	1,137	957

Similar patterns were observed in fungal network complexity (Figure 5B and Table 3). The average degree of fungal networks subsequently decreased from root (15.46) to stem and leaf (stem, 9.58; leaf, 6.26), and then to flower (5.78). Additionally, high values of topological properties (i.e., nodes, edges, positive edges, average clustering coefficient, and total triangles) were found in root (216, 1,670, 1,664, 0.88, and 21,599, respectively), and stem (230, 1,102, 1,089, 0.86, and 5,941, respectively), followed by leaf and flower (leaf: 189, 546, 522, 0.71, and 1,137, respectively; flower: 117, 336, 365, 0.72, and 957, respectively). These data showed that compartment niches subsequently reduced the microbial network complexity and connectivity from root to flower.

### Origin and Transmission Mode of Hemp-Associated Microbiomes

**FIGURE 6:**
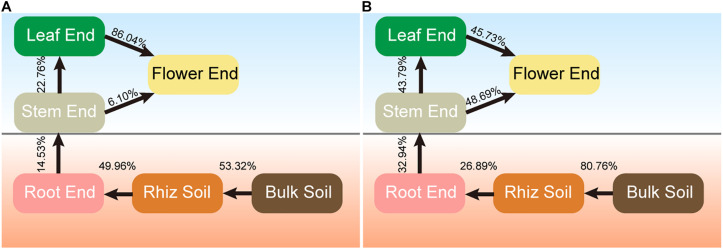
Source model showing the potential sources of hemp associated microbial communities. **(A)** Bacteria. **(B)** Fungi.

Source tracking results showed differences in the sources of bacterial and fungal endophytes (Figure 6). For bacterial communities, 53.32% of rhizobacterial community was derived from the bulk soil community, and 49.96% of root endophytic community was traced to the rhizobacterial community (Figure 6A). The flower endophytic community was mainly from the leaf endophytic community (86.04%).

The majority of the rhizosphere fungal community was traced to the bulk soil community (80.76%), while only 26.89% of root endophytic community was derived from the rhizosphere community (Figure 6B). Approximately 43.79% of the leaf endophytic community was derived from the stem endophytic community, and nearly half of the flower endophytic community was from the stem (48.69%), and leaf (45.73%) communities, respectively. These results indicated that hemp-associated bacterial and fungal endophytes were mainly derived from soil and were gradually filtered in different compartment niches.

## Discussion

Combined with the microbial communities sequencing of soils (bulk soil and rhizosphere soil) and plant compartments (root, stem, leaf, and flower) of four hemp ecotypes cultivated in the same habitat, the present study can be used to directly evaluate the effects of compartment niches and host ecotypes on microbiome assembly and co-occurrence pattern. However, only one type of soil was used as the subject. The effect of other soil types on the microbiome assembly must be analyzed in future experiments. Comeau et al. (2020) compared the rhizosphere, root endosphere, and phyllosphere of three *C. sativa* chemotypes (chemical phenotypes), and strong cultivar-dependent variations in the fungal and bacterial microbiome were found; the distinct secondary metabolites produced by different *C. sativa* cultivars contributed to these strong cultivar-dependent variance. The current results showed that both compartment niches and host ecotypes influenced the microbiome assembly of hemp, and the effect of compartment niches was greater than that of host ecotypes; a similar result was reported previously (Coleman-Derr et al., 2016).

On the basis of the above results, this work focused on the effects of compartment niches on the hemp-associated microbiomes. The alpha diversity was the greatest in soils (bulk and rhizosphere soil), subsequently decreased from root to stem and leaf, and was the lowest in flower. Similar results have been reported in other crops, such as maize (*Zea mays*), wheat (*Triticum aestivum*), and barley (*Hordeum vulgare*); and the results showed that the diversity and network complexity of bacterial communities subsequently increased from endosphere to soil (Xiong et al., 2021). These observations indicate that soil serves as a primary reservoir for plant-associated microbiome, and plant can recruit or filter microbes inhabiting the rhizosphere and endosphere. For grapevine, microbial diversities were greater in belowground niches (i.e., root, root zone, and bulk soil) than in aboveground niches (i.e., leaf, flower, and fruit) (Zarraonaindia et al., 2015). In *Populus*, bacterial/archaeal diversities were greater in soil and root than in leaf and stem, whereas fungal diversities were greater in stem than in leaf or soil (Cregger et al., 2018). Such huge loss in species diversity from soil to plant compartments indicates that only a limited number of microbes could keep a symbiotic lifestyle with host, and is associated with the strong selectivity of plant (Bulgarelli et al., 2012).

Niche differentiation, especially among soil and plant compartments, led to compositional and structural variations in microbial communities among hemp niches. The rhizosphere communities of *C. sativa* across six fields were largely similar to the bulk soil communities, while the root, leaf, and flower communities exhibited distinct compositions (Barnett et al., 2020). The belowground microbiome (rhizosphere, root endosphere) of three *C. sativa* chemotypes differed significantly from the aboveground microbiome (leaves, sweet leaves, and inflorescence) (Comeau et al., 2020). Similar results have been reported in model species *P. deltoides*, in which the microbial community composition in stem and leaf were markedly distinguished from that in rhizosphere soil (Beckers et al., 2017). These compositional differences could be a consequence of host selection. In the present study, Proteobacteria (67.26%) and Cyanobacteria (14.40%) exhibited the highest relative abundance and significant differences among soil and plant compartments; this finding was similar to the phenomenon in cycad (*Cycas panzhihuaensis*) (Zheng and Gong, 2019). *Rhizobium*, *Pseudomonas*, *Bacillus*, and *Sphingomonas* were the top bacterial genera and significantly enriched in aerial samples (*p* < 0.05). *Pseudomonas*, *Sphingomonas*, and *Bacillus* were also dominant in many other plants and known as plant growth-promoting bacteria (Chen et al., 2019). The dominated fungal phyla, Ascomycota (50.59%) and Basidiomycota (15.43%), also exhibited a host effect (*p* < 0.05). Ascomycota was widely distributed in various habitats and showed higher species diversity and faster evolutionary rate than Basidiomycota (Wang et al., 2010). Basidiomycota could maintain soil balance and improve plant productivity in the alpine and temperate grasslands of China (Yang et al., 2019). *Clitopilus*, *Plectosphaerella*, *Alternaria*, *Hypocrea*, and *Mortierella* were the most abundant fungal genera and significantly enriched in belowground endosphere (*p* < 0.05). These core microbial taxa in soil and plant compartments are probably to be vertically transmitted and display species conservatism to a certain degree.

Biomarkers, which are considered as potential keystone taxa, have important and specific functional roles in microbiome assembly and ecosystem functions (Delgado-Baquerizo et al., 2018). According to the LEfSe, Proteobacteria, and Cyanobacteria were potential biomarkers in stem and flower endosphere niches. These two bacterial phyla, which inhabited various types of environments, played an important function in soil and plants, and provided a rich source of inorganic N for plants due to their nitrogen-fixing ability (Prasanna et al., 2009). *Rhizobium*, which had a high potential for nutrient uptake (Flores-Felix et al., 2013), was highly enriched in the root endosphere. Xanthomonadales, Spongiibacteraceae, and Burkholderiales, which had beneficial effects on plant growth and health (Fahlgren et al., 2010), were found to be dominant members of endosphere microbiome. Ascomycota, Sordariomycetes, Dothideomycetes, Pleosporales, and *Penicillium* were dominant in endosphere samples, and these fungal endophytes played an important role in nutrients cycling and the functional coupling of terrestrial ecosystems (Vacher et al., 2016). Additionally, endophytic bacteria (i.e., *Bacillus altitudinis* and *Paenibacillus polymyxa*), and endophytic fungi (i.e., *Aspergillus niger*, *Fusarium moniliforme*, and *Trichoderma viride*) could increase the production of secondary metabolites in medicinal plants (Namdeo et al., 2002; Gao et al., 2015; Song et al., 2017). This report provides empirical evidence for the existence of compartment niche effect of hemp-associated microbiomes, that is, each compartment niche of hemp filters or enriches microbial groups with specific functions.

Network analyses was conducted to explore the interaction patterns of microorganisms in bulk and rhizosphere soils of wheat across North China Plain, and the results showed that wheat rhizosphere soil exhibited less complex topology and more stable co-occurrence pattern than bulk soil (Fan et al., 2018). The links among genera in our network were predominantly positive, and this phenomenon was also observed in the microbial networks of bulk and rhizosphere soils from bark, mosses, and lichens (Aschenbrenner et al., 2017). These results suggested the potential for extensive cooperative interactions among most taxa in their respective micro-environments (Qian et al., 2019). Additionally, bulk and rhizosphere soil exhibited more complex and highly connected bacterial and fungal communities than plant compartments (root, stem, leaf, and flower); this finding was similar to a previous report (Naylor et al., 2017). Nutrients in bulk and rhizosphere soils could attract abundant microorganisms, making these environments one of the most dynamic niches worldwide (Raaijmakers, 2015). Certain topological properties (such as the numbers of nodes, edges, and positive edges as well as average path length) were higher in the root than in other plant compartments. Highly connected architecture could prime the plant immune system for the accelerated activation of defense against pathogens (Lax et al., 2014). Therefore, the root had stronger defense system than other plant compartments.

Source tracking analysis, a useful computational tool that can be used to estimate the proportions of taxa from certain environment, has been extensively used in different fields (Liu et al., 2018). In this study, this analysis was performed to calculate the proportions of endosphere communities derived from soils. The results showed that the bacterial (53.32%) and fungal (80.76%) communities in the rhizosphere soil were mainly derived from bulk soil, and 49.96% of bacterial and 26.89% of fungal communities in the root endosphere were derived from the rhizosphere communities. These results were consistent with the previous literature, which reported that the bulk soil was the main source of microbial communities in rhizosphere soil, and the roots played an important selective role in microbial communities from rhizosphere soil to endophytic communities (Hu et al., 2020). This phenomenon explained the lower alpha diversity in endosphere communities than that in soil communities. The main source of endophytic bacterial (86.04%) and fungal (48.69%) communities in the flower were the leaf and stem, respectively. This finding supported the strong selective effect of compartment niches on endophytes. Understanding the potential sources and environmental process of hemp-associated microbiomes can provide critical information on the interactions among plant, soil, and microbes (Zhang et al., 2017).

## Data Availability Statement

The datasets presented in this study can be found in online repositories. The names of the repository/repositories and accession number(s) can be found below: https://www.ncbi.nlm.nih.gov/, PRJNA690686; https://www.ncbi.nlm.nih.gov/, PRJNA690692.

## Author Contributions

GW analyzed the data and drafted the manuscript. KN performed the wet-lab experiments. HY, SY, and FD collected the samples. GZ analyzed the data and revised the manuscript critically. LD and SC coordinated the study, granted funds, and participated in the drafting and revision of the manuscript. All authors read and approved the final manuscript.

## Conflict of Interest

HY, SY, and FD are employed by Yunnan Industrial Investment Group, Yunnan Hemp Seed Industry Co., Ltd. The remaining authors declare that the research was conducted in the absence of any commercial or financial relationships that could be construed as a potential conflict of interest.

## Publisher’s Note

All claims expressed in this article are solely those of the authors and do not necessarily represent those of their affiliated organizations, or those of the publisher, the editors and the reviewers. Any product that may be evaluated in this article, or claim that may be made by its manufacturer, is not guaranteed or endorsed by the publisher.

## Footnotes

1
http://mkweb.bcgsc.ca/tableviewr/visualize/
