# Supplementary material for: Compartment Niche Shapes the Assembly and Network of Cannabis sativa-Associated Microbiome
Source: Front Microbiol. 2021 Oct 5;12:714993. doi: 10.3389/fmicb.2021.714993 (PMC8524047; doi:10.3389/fmicb.2021.714993)
Supplement: Supplementary file 1 [file Data_Sheet_1.doc]

**Title: Compartment niche shapes assembly and network of *Cannabis*** ***sativa* -associated microbiome**

**Running title: Assembly and network of *C.*** ***sativa* -associated microbiome**

*Guangfei Wei^1^, Kang Ning^1^, Guozhuang Zhang^1^, Haibin Yu^2^, Shuming Yang^2^, Fei Dai^2^, Linlin Dong^1,*^ and Shilin Chen^1,*^*

^1^ *Key Laboratory of Beijing for Identification and Safety Evaluation of Chinese Medicine, Institute of Chinese Materia Medica, China Academy of Chinese Medical Sciences, Beijing 100700, China*

*^2^ Yunnan Industrial Investment Group, Yunnan Hemp Seed Industry CO., LTD.*

**E-mail addresses:**

Guangfei Wei: [gfwei@icmm.ac.cn](mailto:dll_aaa@163.com)

Kang Ning: cjdgtq@163.com

Guozhuang Zhang: [zgz123zgz@163.com](mailto:zgz123zgz@163.com)

Haibin Yu: [yhbcn@126.com](mailto:yhbcn@126.com)

Shuming Yang: [865513507@qq.com](mailto:865513507@qq.com)

Fei Dai: 66374603@qq.com

*** Corresponding author**

TEL: (+86) 18911917789; fax: (+86) 1062899776; email: [lldong@icmm.ac.cn](mailto:lldong@icmm.ac.cn)

*** *Corresponding author**

TEL: (+86) 1057203877; fax: (+86) 1062899776; email: slchen@icmm.ac.cn

Present address: No.16 Nanxiaojie, Dongzhimennei Ave. Beijing 100700, China.

**Supporting Information**

**A. Supporting Figures**

**Figure S1** Flower plots of hemp associated microbial communities.

**B. Supporting Tables**

**Table S1** The information of hemp ecotypes.

**Table S2** Bacterial and fungal sequences numbers of hemp associated microbiomes.

**Table S3** Alpha diversity of hemp associated microbiomes.

**Fig. S1** Flower plots of hemp associated microbial communities. (A) Bacteria. (B) Fungi. Ct, Rs, Ro, St, Le, and Fl represent bulk soil, rhizosphere soil, root endosphere, stem endosphere, leaf endosphere and flower endosphere, respectively.


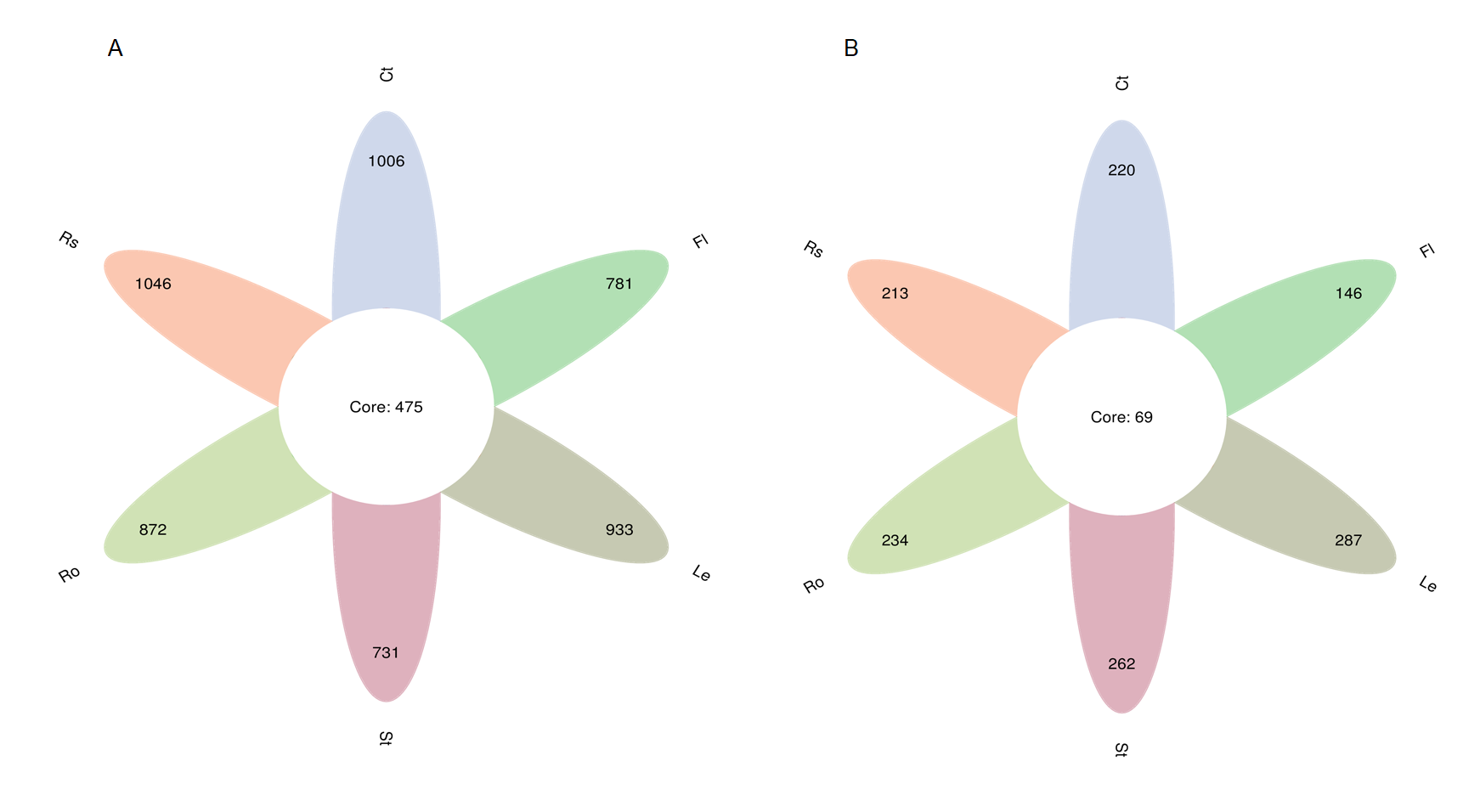


**Table S1** The information of industrial hemp ecotypes.

| Name | Ecotypes | Types | Characterisics |
| --- | --- | --- | --- |
| GS | Gansuqingshui | Seed type | Gansuqingshui, early-maturing, strong-resistance, and high seed yield |
| YN | Yunma No. 1 | Seed and stem compatible type | fast growing, tall plants, high fiber tield and high seed rate |
| MG | Yunmaza No. 3 | Fiber and medicine compatible type | strong resistance to dense planting and high content of cannabinoid |
| HLJ | Huoma No. 1 | Fiber type | high fiber, high quality, disease resistance, lodging resistance, salt and alkali resistance |

**Table S2** Bacterial and fungal sequences numbers of hemp associated microbiomes. GS, HLJ, MG and YN represent Gansuqingshui, Huoma No. 1, Yunmaza No. 1, and Yunnan No. 1, respectively. Ct, Rs, Ro, St, Le, and Fl represent bulk soil, rhizosphere soil, root endosphere, stem endosphere, leaf endosphere and flower endosphere, respectively. -1,-2 and -3 represent three replicates in one site.

| Samples | Bacterial sequences | Fungal sequences |
| --- | --- | --- |
| HLJ_Ct_1 | 87430 | 81767 |
| HLJ_Ct_2 | 66543 | 81120 |
| HLJ_Ct_3 | 85133 | 84522 |
| HLJ_Rs_1 | 84771 | 82679 |
| HLJ_Rs_2 | 81736 | 85071 |
| HLJ_Rs_3 | 87212 | 87510 |
| HLJ_Ro_1 | 87213 | 86381 |
| HLJ_Ro_2 | 83670 | 85993 |
| HLJ_Ro_3 | 84627 | 80564 |
| HLJ_St_1 | 83322 | 85404 |
| HLJ_St_2 | 84249 | 86671 |
| HLJ_St_3 | 83608 | 83037 |
| HLJ_Le_1 | 87223 | 85957 |
| HLJ_Le_2 | 87805 | 84552 |
| HLJ_Le_3 | 87024 | 86531 |
| HLJ_Fl_1 | 73734 | 82237 |
| HLJ_Fl_2 | 80399 | 82119 |
| HLJ_Fl_3 | 86298 | 80760 |
| MG_Ct_1 | 87597 | 80628 |
| MG_Ct_2 | 80321 | 86603 |
| MG_Ct_3 | 84717 | 85862 |
| MG_Rs_1 | 83575 | 80986 |
| MG_Rs_2 | 125752 | 82273 |
| MG_Rs_3 | 80822 | 81765 |
| MG_Ro_1 | 87596 | 84626 |
| MG_Ro_2 | 86328 | 85701 |
| MG_Ro_3 | 87045 | 81408 |
| MG_St_1 | 86844 | 85049 |
| MG_St_2 | 81869 | 86259 |
| MG_St_3 | 86716 | 82367 |
| MG_Le_1 | 87896 | 86055 |
| MG_Le_2 | 86722 | 85878 |
| MG_Le_3 | 82298 | 81739 |
| MG_Fl_1 | 84688 | 86801 |
| MG_Fl_2 | 80514 | 86419 |
| MG_Fl_3 | 87523 | 84082 |
| YN_Ct_1 | 82294 | 86049 |
| YN_Ct_2 | 80882 | 82833 |
| YN_Ct_3 | 82499 | 80562 |
| YN_Rs_1 | 80965 | 87170 |
| YN_Rs_2 | 64424 | 83930 |
| YN_Rs_3 | 67885 | 86765 |
| YN_Ro_1 | 81635 | 84115 |
| YN_Ro_2 | 84641 | 81596 |
| YN_Ro_3 | 84239 | 84025 |
| YN_St_1 | 82964 | 85780 |
| YN_St_2 | 83473 | 84652 |
| YN_St_3 | 87906 | 85092 |
| YN_Le_1 | 80196 | 84099 |
| YN_Le_2 | 83328 | 87500 |
| YN_Le_3 | 85502 | 87191 |
| YN_Fl_1 | 80166 | 80250 |
| YN_Fl_2 | 85415 | 85169 |
| YN_Fl_3 | 85789 | 82933 |
| GS_Ct_1 | 124796 | 82337 |
| GS_Ct_2 | 61945 | 87336 |
| GS_Ct_3 | 85580 | 84250 |
| GS_Rs_1 | 82979 | 84874 |
| GS_Rs_2 | 80416 | 83112 |
| GS_Rs_3 | 83468 | 84640 |
| GS_Ro_1 | 82599 | 87516 |
| GS_Ro_2 | 86635 | 83122 |
| GS_Ro_3 | 87575 | 81739 |
| GS_St_1 | 81282 | 87678 |
| GS_St_2 | 85611 | 81363 |
| GS_St_3 | 82042 | 81717 |
| GS_Le_1 | 82120 | 84937 |
| GS_Le_2 | 81606 | 83142 |
| GS_Le_3 | 85955 | 85596 |
| GS_Fl_1 | 83198 | 87378 |
| GS_Fl_2 | 81300 | 85155 |
| GS_Fl_3 | 82737 | 83051 |
| Total | 6056867 | 6066030 |

**Table S3 Alpha diversity of hemp associated microbiomes.**

| Compartments | Bulk soil  (%) | Significant difference | Rhizosphere soil  (%) | Significant difference | Root endosphere  (%) | Significant difference | Stem endosphere  (%) | Significant difference | Leaf endoshere  (%) | Significant difference | Flower endosphere  (%) | Significant difference | *P*-value |
| --- | --- | --- | --- | --- | --- | --- | --- | --- | --- | --- | --- | --- | --- |
| Bacterial chao 1 | 1560.73±624.99 | a | 1845.69±807.81 | a | 1315.16±343.50 | ab | 581.42±340.48 | c | 829.01±509.35 | bc | 595.70±330.34 | c | 1.9×10^-8^ |
| Bacterial shannon | 8.08±2.61 | a | 8.08±3.42 | a | 8.52±0.44 | a | 3.99±2.85 | b | 4.95±2.38 | b | 3.77±2.04 | b | 5.4×10^-7^ |
| Fungal chao 1 | 258.03±57.61 | a | 252.57±59.94 | ab | 186.35±66.33 | bc | 108.93±63.19 | de | 144.76±66.94 | cd | 53.14±24.21 | e | 1.6×10^-13^ |
| Fungal shannon | 4.53±0.47 | a | 4.38±0.39 | a | 3.38±0.85 | b | 2.32±1.31 | c | 3.15±1.01 | bc | 1.15±0.56 | d | 3.3×10^-15^ |
